# Mating Patterns and Postzygotic Barriers in a Hybrid Swarm of Two Closely Related Pigeon Species

**DOI:** 10.1002/ece3.73455

**Published:** 2026-04-15

**Authors:** Jin‐yong Kim, Seung‐Gu Kang, Jongmin Yoon

**Affiliations:** ^1^ Research Center for Endangered Species National Institute of Ecology Yeongyang South Korea

**Keywords:** assortative mating, feral pigeons, hill pigeons, hybrid swarm, reproductive barrier

## Abstract

Hybrid zones provide a valuable framework for understanding evolutionary pathways. We investigated mating patterns and reproductive barriers using captive individuals derived from a natural hybrid swarm between hill pigeons ( *Columba rupestris* ) and feral pigeons ( *C. livia* ), maintained under controlled conditions. Both parental taxa exhibited significant assortative mating. The observed mating structure suggests that morphological similarity, mate availability, social interactions, and genetic relatedness may jointly shape pairing patterns. Reproductive performance was assessed at three stages—clutch size, hatching, and fledging—across four cross types (hill pigeon, feral pigeon, within‐hybrid, and heterotypic cross types). Clutch size did not differ among cross types. Hatchling and fledgling mortality in within‐hybrid crosses and heterotypic crosses did not differ from that in hill pigeon conspecific crosses. Within‐hybrid crosses and heterotypic crosses had higher hatchling mortality than feral pigeon conspecific crosses, and only heterotypic crosses showed higher fledgling mortality. Between parental taxa, hill pigeon conspecific crosses exhibited higher hatchling mortality than feral pigeon conspecific crosses. These results suggest asymmetric postzygotic isolation that is most evident in comparisons involving feral pigeons, with implications for reinforcement of conspecific mate choice in feral pigeons and continued directional introgression into hill pigeons.

## Graphical abstract

Based on the observed mating structure and reproductive outcomes, hybrid index values of the resulting offspring shifted toward the feral pigeon end. This pattern indicates ongoing introgression of feral‐derived alleles into the hybrid population.

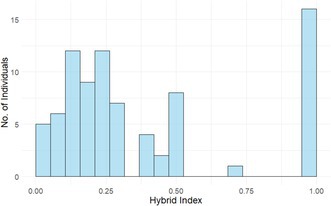

## 1 Introduction

Hybrid zones are geographic regions where genetically distinct populations meet and interbreed, resulting in at least some offspring of mixed ancestry (Barton and Hewitt 1985, 1989; Harrison 1993). These zones often emerge in ecotones or transitional habitats where the ranges of previously isolated populations overlap, and may be maintained by a balance between dispersal, local environmental conditions that permit coexistence, and selection against hybrids (Barton and Hewitt 1985). Hybrid zones are considered active sites of evolutionary change, functioning either as sources of new recombinant types (i.e., new species) or as regions where selection against hybridization promotes strong prezygotic and postzygotic barriers to gene exchange (Harrison 1993). In the latter case, hybrid zones can be arenas for reinforcement, where natural selection acts to strengthen reproductive isolation by favoring traits that reduce the likelihood of interspecific mating (Servedio and Noor 2003).

According to the biological species concept (Mayr 1942), species are defined as groups of actually or potentially interbreeding natural populations that are reproductively isolated from other such groups. Reproductive isolation is therefore central to the maintenance of species boundaries, as it prevents or limits gene flow between divergent populations. This isolation can occur through a variety of mechanisms, broadly classified as premating prezygotic (e.g., habitat, temporal, behavioral, or mechanical isolation), postmating prezygotic (e.g., gametic incompatibility), and postzygotic (e.g., reduced hybrid viability or fertility) barriers (Servedio and Noor 2003; Hoskin et al. 2005). In addition, nonrandom mating patterns, such as assortative mating, can act as mechanisms contributing to premating prezygotic isolation (Servedio and Noor 2003). Within the framework of reinforcement theory, when hybrids exhibit significantly lower fitness compared to parental genotypes, natural selection acts to reduce the production of such unfit hybrids, thereby promoting the evolution of additional reproductive isolation mechanisms (Dobzhansky 1940; Muller 1942; Servedio and Noor 2003). Liou and Price (1994) show that even in the presence of gene flow, reinforcement can lead to the evolution of assortative mating preferences that reduce interspecific mating. In their model, the existence of postzygotic incompatibilities provides the selective pressure necessary for the evolution of mating discrimination, thereby minimizing the formation of hybrids with reduced fitness.

The global expansion of the feral pigeon ( *Columba livia* var. *domestica*) populations–largely driven by human introduction and release–together with their adaptability to diverse environments, suggests that the threat of hybridization is not only persistent but may be escalating. Notably, hybridization between the hill pigeon ( *Columba rupestris* ) and feral pigeon occurs frequently in areas where their geographic ranges overlap (Baptista et al. 2020; Kim, Hwang, et al. 2022). Hill pigeons are distributed across Central and East Asia, inhabiting rocky cliffs and arid mountainous landscapes from Mongolia and China to parts of the Korean Peninsula. South Korea lies at the easternmost edge of the hill pigeon's range, and the species has been designated Endangered Species (Class II) nationally due to sustained population declines associated with hybridization. In contrast, feral pigeons, the feralized form of the Rock pigeon ( *Columba livia* ), are now ubiquitous globally, largely due to human‐mediated translocation and artificial selection for traits such as color (e.g., pale blue‐bar and darker melanic/spred morphs, as well as red and white), size, homing ability, and tameness (Johnston and Janiga 1995; Galdames et al. 2025). Feralization has resulted in an immunologically and phenotypically diverse array of pigeon breeds, many of which retain the ability to interbreed with their wild congeners (Galdames et al. 2025). The introduction and establishment of feral pigeons in urban, rural, and even natural habitats have led to increasing spatial overlap with hill pigeon populations. This spatial convergence creates opportunities for interbreeding and has been documented to produce viable hybrid offspring, raising concerns about genetic introgression and the potential erosion of hill pigeons' gene pool (Kim, Eo, et al. 2022).

Uiryeong County, located in South Korea, was historically part of the native range of the hill pigeon. However, the current population in this region comprises a small number of hill pigeons and feral pigeons, and a predominance of hybrid individuals (Kim, Eo, et al. 2022). Some individuals exhibit morphological traits characteristic of F1 hybrids−such as a gray rather than white rump, poorly defined black bars at the bases of the greater and third wing coverts, and a faint or indistinct white terminal band on the central tail feathers—while others resemble hill pigeons but do not fully conform to the species' typical phenotype (Kang et al. 2022). Genetic hybrid marker (Kim, Hwang, et al. 2022) testing indicated that all later‐generation hybrid individuals captured in this area possess genotypes intermediate between hill pigeons and F1 hybrids (Table S1). This pattern suggests that the population likely originated from initial hybridization between hill pigeons and feral pigeons, followed by repeated backcrossing with hill pigeons over time. The current composition of the population, in which most individuals exhibit a mosaic of hybrid traits, reflects an extended period of hybridization consistent with the characteristics of a hybrid swarm (Allendorf et al. 2001). Hybrid swarms, typically arise following anthropogenic disturbances (e.g., species introductions and habitat alteration) and demographic imbalance that bring previously isolated lineage into contact (Allendorf et al. 2001). When populations differ in abundance, individuals from the rarer population may mate disproportionately with individuals from the more common population, generating many hybrids; depending on hybrid fitness, this can result in a hybrid swarm or, alternatively, the extinction of the rarer population (Liou and Price 1994; Allendorf et al. 2001). Nevertheless, studying these populations provides a valuable framework for understanding the persistence and evolutionary potential (i.e., the longer‐term consequences of hybridization) of hybrid forms, because hybrid swarms may act as reservoirs of genetic variation, facilitate ecological novelty, and—under certain selection regimes—contribute to either speciation or genetic homogenization (Yakimowski and Rieseberg 2014).

Our study aimed to investigate a stage at which hybridization had already occurred between hill and feral pigeon populations following the human‐mediated introduction of feral pigeons, resulting in the formation of a hybrid swarm. Using a controlled captive‐breeding design with individuals sampled from a natural hybrid swarm, we examined the mating patterns and reproductive outcomes among cross types with distinct genetic backgrounds. To achieve this goal, we tested the following hypotheses: (1) mating is nonrandom, with bias toward within−cross−type matings (assortative mating), and (2) heterotypic crosses exhibit significantly higher mortality rate compared to conspecific crosses. By doing so, we aim to better understand how hybrid swarms maintain their structure and what evolutionary dynamics (e.g., nonrandom mating and selection against hybrids) influence the potential outcomes of prolonged hybridization.

## 2 Material and Methods

### 2.1 Study Design

**FIGURE 1:**
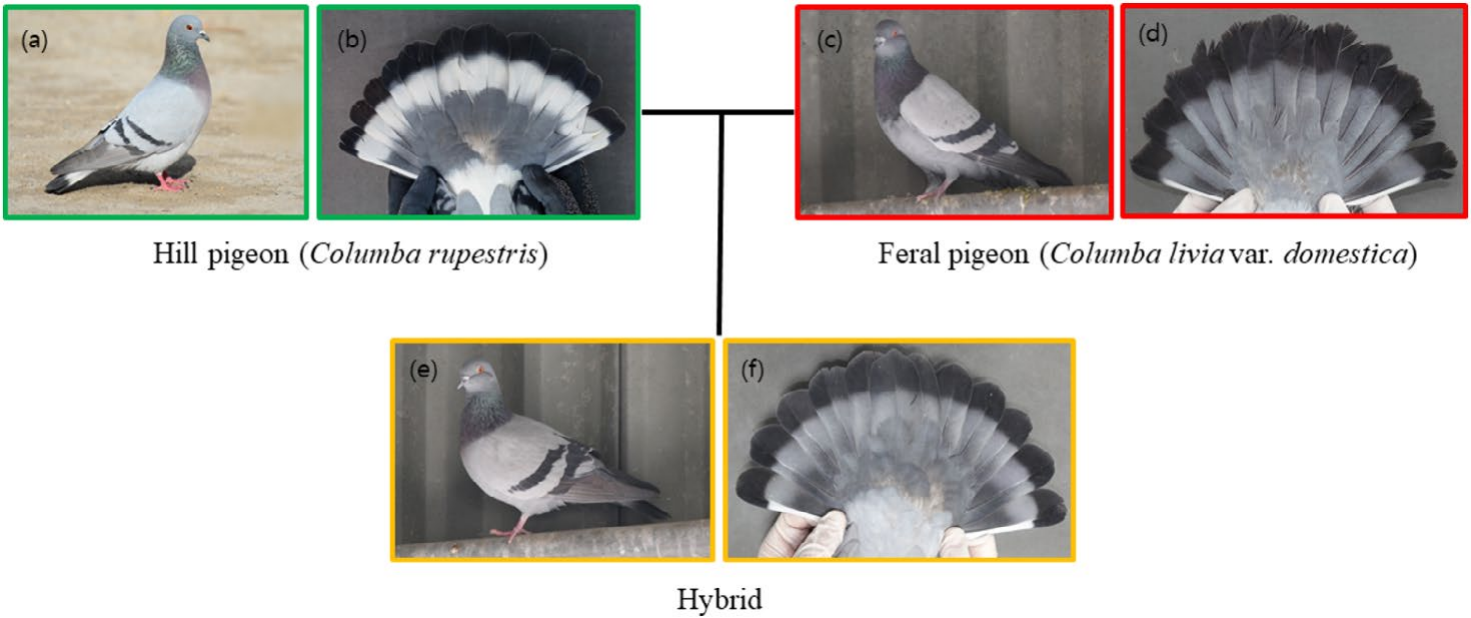
Comparative external morphology of the hill pigeon ( *Columba rupestris* ), feral pigeon (*Columba livia var. domestica*), and their hybrid. (a, b) Hill pigeon: Body appearance (a) and tail pattern (b). (c, d) Feral pigeon: Body appearance (c) and tail pattern (d). (e, f) Hybrid: body appearance (e) and tail pattern (f).

**FIGURE 2:**
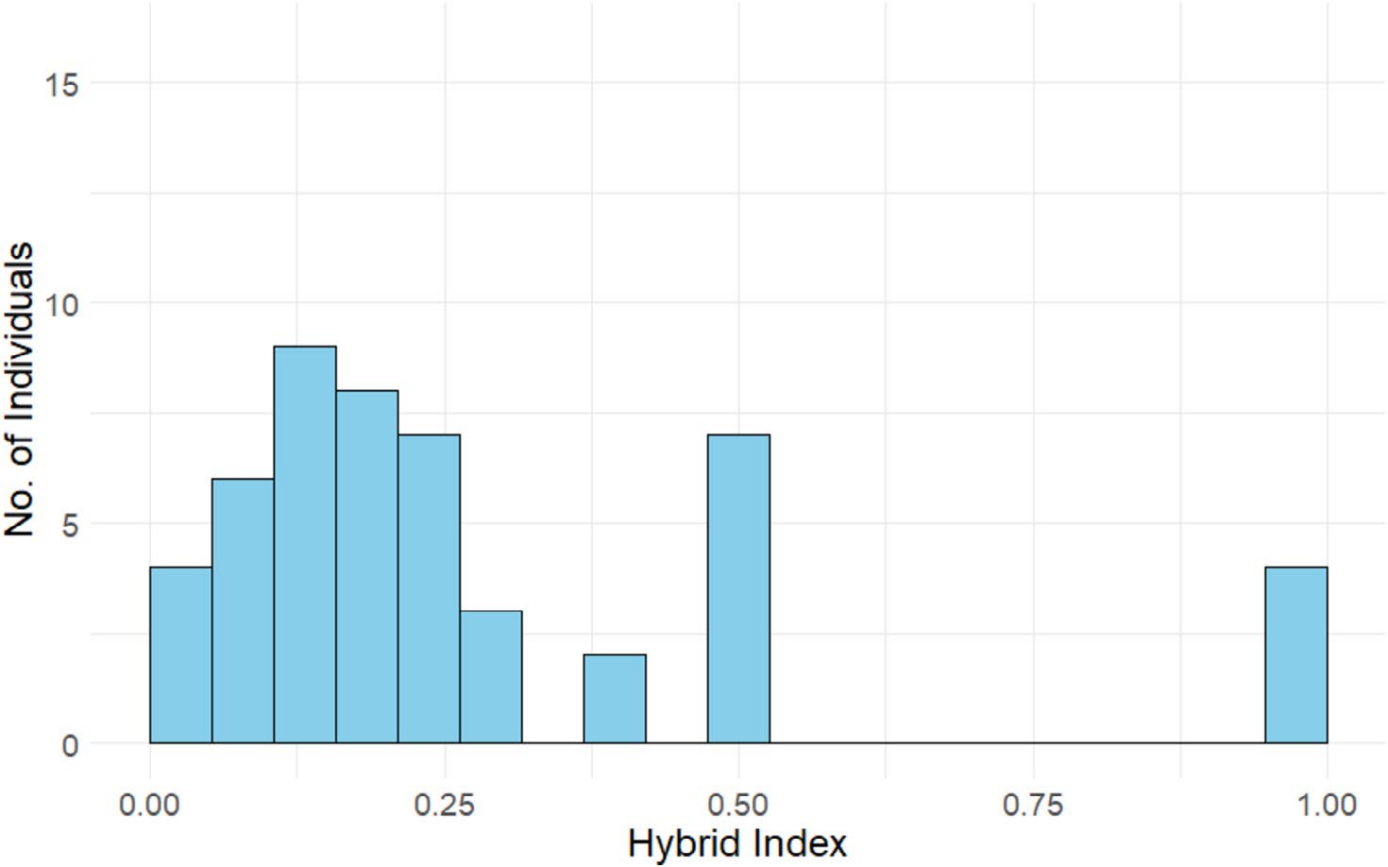
Distribution of hybrid index scores for experimental individuals used in mating and reproduction in this study.

Currently, Korean populations of hill pigeons are distributed in Gurye and Yeoncheon Counties, South Korea (Kang et al. 2022). Historically, they were found in Gurye, Goheung, and Uiryeong Counties, but the Goheung population is locally extinct, and most of the Uiryeong population has become hybridized due to interbreeding with feral pigeons (Kim, Eo, et al. 2022). The individuals to be used in the experiment were mostly captured from wild habitats between 2019 and 2021, whereas F1 hybrids were bred at the Endangered Species Restoration Center of the National Institute of Ecology. The phenotypic differences among the hill pigeon, feral pigeon, and their hybrids are illustrated in Figure 1. Individuals were assigned to genetic categories based on their genetic background. In this study, we classified birds into four groups: hill pigeons (HP; Gurye: ♂ 2, ♀ 2), Later‐generation hybrids (LG; Uiryeong: ♂ 18, ♀ 17), First generation hybrids (F1; ♂ 5, ♀ 2), and feral pigeons (FP: ♂ 2, ♀ 2), all maintained under the same controlled conditions. The degree of hybridization and the presence of pure species were assessed in all individuals using genetic hybrid markers developed in a previous study (Kim, Hwang, et al. 2022). A total of 8 Indel markers were developed previously, which exhibit different base pair (bp) patterns in hill and feral pigeons. F1 hybrids show double bands, which can be visually confirmed through agarose gel electrophoresis. Based on these markers, hill pigeons were assigned a score of 0, feral pigeons a score of 1, and F1 hybrids a score of 0.5. The average value was calculated and expressed as the hybrid index. The hybrid index values were 0 for HP, 0.01–0.49 for LG, 0.5 for F1, and 1 for FP (Table S1). The experimental individuals used in this study exhibited a typical form of a hybrid swarm skewed toward the hill pigeon (Figure 2; Table S1).

### 2.2 Captive Condition and Monitoring

All mating and reproductive outcomes data across three sequential stages—clutch size, hatching, and fledging—were collected in a controlled captive environment that served as an artificial hybrid zone, ensuring that individuals from all cross types had equal opportunity to form pairs without spatial bias. Data were collected from March 2023 to July 2024. The size of the aviary was 11.5 m × 11.5 m × 5.7 m (floor area = 131.81 m^2^). Twenty artificial nest boxes for breeding and perches for resting were provided; nest boxes were installed along one wall at an elevated position to reduce potential disturbance during routine husbandry. Food (mixed grain) and water were continuously supplied to ensure they were never in short supply, and cuttlefish bone was provided as a calcium source. All individuals were banded with plastic color rings and metal numbered rings on their tarsus. Video cameras were used to confirm the breeding pairs during incubation. Pigeons typically form stable social pair bonds during breeding in captivity, which supports the use of incubation‐based pair identification as the unit of analysis (Patel and Siegel 2005). The clutch size for hill and feral pigeons is a maximum of 2 eggs, and they typically lay 1 egg per day. Therefore, the number of eggs, hatched chicks, and fledglings, along with their dates, were recorded every 2 days. The survival of fledglings was determined by their survival for at least 3 months after fledging.

### 2.3 Statistical Analyses

To investigate nonrandom and directional mating, we analyzed 26 unique breeding pairs (ordered male–female combinations). Under the null hypothesis of random mating, we calculated the expected frequency of each possible male–female combination using class sample sizes, assuming that pairing probabilities are proportional to class frequencies:

(1) $$E\left(i,j\right)=\left(\frac{N_{\text{male},i}}{N_{\text{total males}}}\right)\times \left(\frac{N_{\text{female},j}}{N_{\text{total females}}}\right)\times N_{\text{total pairs}}$$

where *E*(*i*, *j*) is the expected number of pairs between male class *i* and female class *j* and *N*_total pairs_ = 26.

We then conducted a permutation‐based analysis to examine deviations for each specific pairing type using permuted 1000 times to build a null distribution of pairing frequencies for each class combination. The *Z*‐score was calculated to determine how many standard deviations the observed value deviated from the permutation‐based expectation. The *Z*‐score is approximately normally distributed with a mean of 0, and values exceeding ±1.65 were considered to depart significantly from random‐mating expectations. All statistical analyses were conducted in R (version 4.3.0; R Core Team 2023).

Clutch size (number of eggs per breeding attempt; maximum = 2) was compared among cross types using a Kruskal–Wallis rank‐sum test because the response is discrete and not well approximated by a normal distribution. Cross types were defined as follows: (1) conspecific crosses—HP–HP (*n* = 6) and FP–FP (*n* = 10); (2) Within‐hybrid crosses—LG–LG (*n* = 42) and F1–F1 (*n* = 2); and (3) heterotypic crosses—LG–F1 (*n* = 4), F1–LG (*n* = 17), FP–LG (*n* = 2), and FP–F1 (*n* = 2). Then *n* values refer to the number of breeding attempts (mating trial; total *N* = 85). We analyzed the effect of cross type on hatchling and fledgling mortality using generalized linear mixed models (GLMMs) with a binomial error structure and logit link. Model structure was specified a priori based on the study hypotheses and the data‐generating process. Cross type as the primary fixed effect it directly represents differences among genetic backgrounds and is central to our prediction about postzygotic costs, whereas breeding‐pair identity was included as a random intercept to account for non‐independence due to repeated breeding attempts and multiple offspring outcomes contributed by the same pair. Cross type (as defined above) was included as the main fixed effect, and breeding‐pair identity was included as a random effect in all models to account for repeated measures. The response variable in each binomial GLMM was specified using the cbind function in R (version 4.3.0; R Core Team 2023), which combines the number of failures (e.g., hatchling_dead) and successes (e.g., hatchling_alive) for each observation into a two‐column matrix (Kuznetsova et al. 2017; Zamora‐Marín et al. 2025). The mortality rate at each developmental stage was calculated as the proportion of failures to the total number of individuals at risk. Stage‐specific mortality was calculated as the proportion of failures among individuals at risk each developmental stage. Hatchling mortality was calculated among hatched chicks (hatchling_dead/(hatchling_dead + hatcling_alive)), and fledgling mortality was calculated among fledgings at risk (fledgling_dead/(fledgling_dead + fledgling_alive)). We present these outcomes as mortality (rather than success) to emphasize the postzygotic costs associated with hybridization in this hybdrid‐swarm system; stage‐specific success is the complement of mortality for the corresponding stage (success = 1—mortality). This approach is consistent with the response‐variable structure used in binomial GLMMs. For all post‐laying stages, zero values were retained as biologically meaningful outcomes (e.g., eggs hatched but no chicks fledged). The binomial model then estimates the log odds of mortality based on these proportions. All models were fitted using the glmmTMB package in R (version 4.3.0; R Core Team 2023). We estimated model coefficient uncertainty with a nonparametric bootstrap approach (5000 replicates), extracting percentile‐based 95% confidence intervals and bootstrap *p*‐values for each fixed effect. Estimated marginal means (predicted probabilities on the response scale) for each cross type were obtained using the emmeans package. Pairwise contrasts among cross types were summarized using (i) the absolute difference in predicted mortality, Δ*p* = *p*A − *p*B (risk difference, in probability units), and (ii) the odds ratio (OR) for A relative to B, defined as OR = [*p*A/(1 − *p*A)]/[*p*B/(1 − *p*B)]. Contrasts were evaluated on the model scale (logit), but are presented as probabilities, Δ*p* and OR for interpretability. To visualize the results, we produced bar plots displaying the predicted mortality rate (with 95% confidence intervals) for each cross type at each stage, with observed raw mortality rates for each breeding pair shown as overlaid jittered points. All figures were created in the ggplot2 package.

## 3 Results

### 3.1 Mating Patterns

Across the 26 unique breeding pairs, conspecific matings showed significant deviations from random mating expectations, indicating strong assortative mating tendencies (Table 1). For example, matings within hill pigeons (HP♂–HP♀) were elevated (Observed = 1; Expected = 0.17; *Z* = 4.467). Similarly, matings within feral pigeons (FP♂–FP♀) were significantly more frequent than expected (Observed = 2; Expected = 0.17; *Z* = 3.726). Across heterotypic matings (LG♂–F1♀, F1♂–LG♀, FP♂–LG♀, and FP♂–F1♀), none deviated significantly from random expectation (Table 1).

**TABLE 1:** Observed and random‐mating expected frequencies of male–female class combination for hill pigeons, feral pigeons, first‐generation hybrids, and later‐generation hybrids, with permutation‐based *Z*‐scores from 1000 randomizations.

Male–female	Observed	Expected	Permutation mean	Permutation SD	*Z* residual
HP♂–HP♀	1	0.17	0.04	0.19	4.467*
LG♂–LG♀	14	12.81	11.56	1.09	1.093
LG♂–F1♀	1	1.51	1.71	0.81	−0.623
F1♂–LG♀	5	3.56	4.57	0.96	1.507
F1♂–F1♀	1	0.42	0.73	0.70	0.831
FP♂–LG♀	1	1.42	3.10	0.79	−0.533
FP♂–F1♀	1	0.17	0.45	0.59	1.400
FP♂–FP♀	2	0.17	0.30	0.49	3.726*

*Note:* *Z* residuals marked with * are statistically significant at *p* ≤ 0.05 (|*Z*| > 1.65).

Abbreviations: F1, first‐generation hybrid; FP, feral pigeon; HP, hill pigeon; LG, later‐generation hybrid.

### 3.2 Reproductive Performance

**FIGURE 3:**
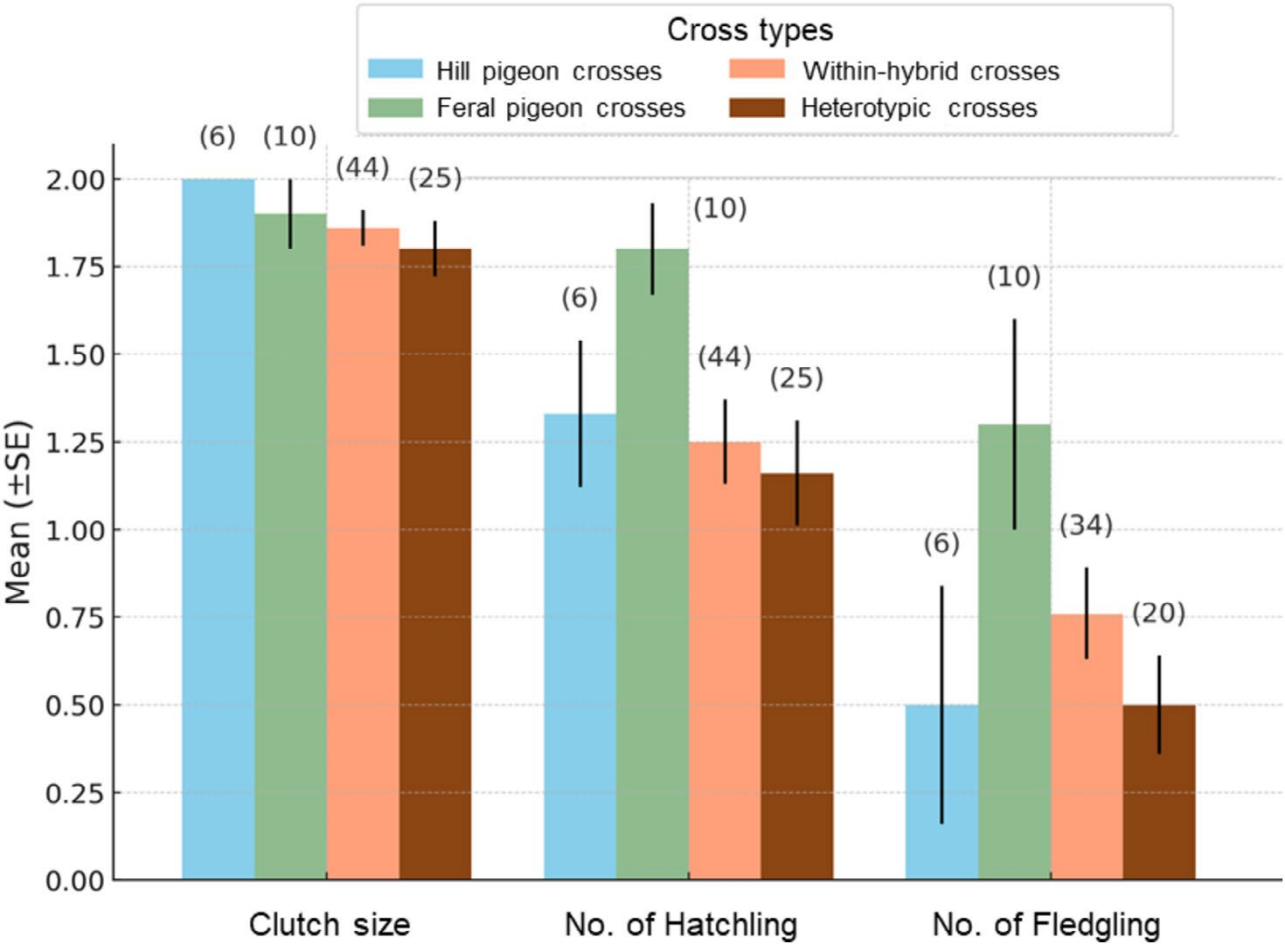
Mean clutch size, hatching, and fledging survival across four cross types.

**FIGURE 4:**
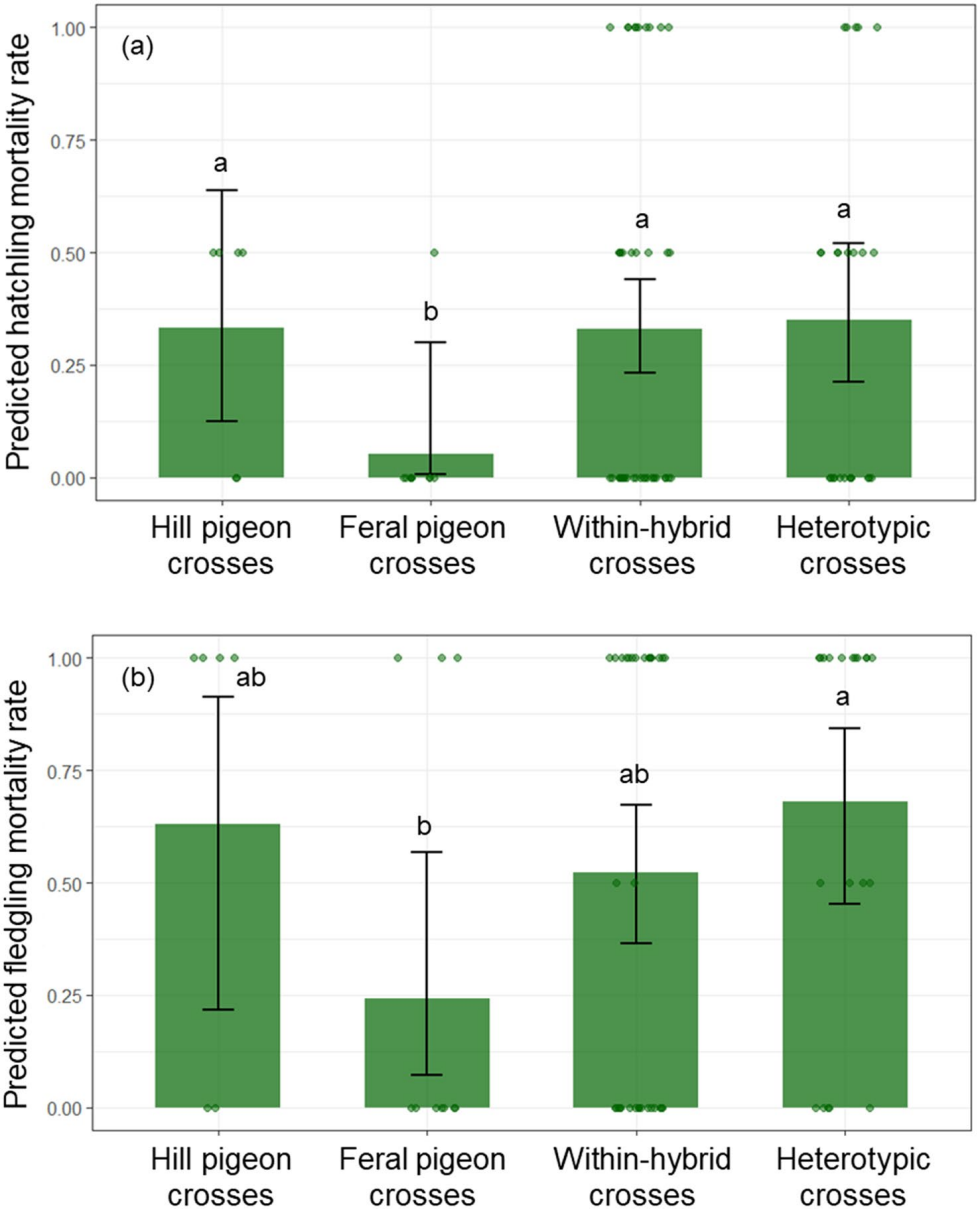
Predicted mortality rates at different breeding stages by cross types, based on 5000 bootstrap replicates. (a) Predicted hatchling mortality rate; (b) Predicted fledgling mortality rate. The error bars represent the 95% bootstrap confidence intervals, and the dots represent the observed mortality rates for breeding pairs. Different letters indicate significant differences among cross types (*p* < 0.05).

Preliminary comparisons of mean reproductive outcomes across cross types are shown in Figure 3. Clutch size did not differ among cross types (Kruskal–Wallis test: *χ* ^2^ = 1.826, *p* = 0.609). We investigated stage‐specific mortality (hatchling and fledgling) across cross types using binomial GLMMs (Table 2; Figure 4). Using hill pigeon conspecific crosses (hill × hill) as the reference, hatchling mortality did not differ from within‐hybrid crosses (Δ*p* = 0.003; OR = 1.01; *p* = 0.809) or heterotypic crosses (Δ*p* = −0.019; OR = 0.92; *p* = 0.839). Similarly, fledging mortality did not differ between hill pigeon conspecific crosses and either within‐hybrid crosses (Δ*p* = 0.110; OR = 1.57; *p* = 0.584) or heterotypic crosses (Δ*p* = −0.049; OR = 0.80; *p* = 0.743). In contrast, feral pigeon conspecific crosses (feral × feral) showed markedly lower hatchling mortality than both within‐hybrid crosses (Δ*p* = −0.278; OR = 0.11; *p* = 0.002) and heterotypic crosses (Δ*p* = −0.300; OR = 0.10; *p* = 0.002). At the fledging stage, feral pigeon conspecific crosses also exhibited lower mortality than heterotypic crosses (Δ*p* = −0.439; OR = 0.15; *p* = 0.007), whereas the difference between feral pigeon conspecific crosses and within‐hybrid crosses was not statistically significant (Δ*p* = −0.280; OR = 0.29; *p* = 0.072). Finally, hill pigeon conspecific crosses had higher hatchling mortality than feral pigeon conspecific crosses (Δ*p* = 0.281; OR = 9.25; *p* = 0.021), while the corresponding difference at the fledging stage was not significant (Δ*p* = 0.390; OR = 5.39; *p* = 0.104).

**TABLE 2:** Predicted stage‐specific mortality (probability; 95% bootstrap CI) and pairwise contrasts among cross types from binomial GLMMs.

Stage	Cross type (A vs. B)	Predicted mortality A (prob, 95% CI)	Predicted mortality B (prob, 95% CI)	Δ*p* (A − B)	OR (A/B)	*p*
Hatching	Hill pigeon crosses vs. Feral pigeon crosses	0.332 (0.0123–0.637)	0.051 (0.007–0.299)	0.281	9.25	0.021*
	Hill pigeon crosses vs. Within‐hybrid crosses	0.332 (0.0123–0.637)	0.329 (0.234–0.441)	0.003	1.01	0.809
	Hill pigeon crosses vs. Heterotypic crosses	0.332 (0.0123–0.637)	0.351 (0.212–0.520)	−0.019	0.92	0.839
	Feral pigeon crosses vs. Within‐hybrid crosses	0.051 (0.007–0.299)	0.329 (0.233–0.441)	−0.278	0.11	0.002*
	Feral pigeon crosses vs. Heterotypic crosses	0.051 (0.007–0.299)	0.351 (0.212–0.520)	−0.300	0.10	0.002*
Fledging	Hill pigeon crosses vs. Feral pigeon crosses	0.631 (0.218–0.913)	0.241 (0.071–0.568)	0.390	5.39	0.104
	Hill pigeon crosses vs. Within‐hybrid crosses	0.631 (0.218–0.913)	0.521 (0.364–0.674)	0.110	1.57	0.584
	Hill pigeon crosses vs. Heterotypic crosses	0.631 (0.218–0.913)	0.680 (0.453–0.844)	−0.049	0.80	0.743
	Feral pigeon crosses vs. Within‐hybrid crosses	0.241 (0.071–0.568)	0.521 (0.364–0.674)	−0.280	0.29	0.072
	Feral pigeon crosses vs. Heterotypic crosses	0.241 (0.071–0.568)	0.680 (0.453–0.844)	−0.439	0.15	0.007*

*Note:* Confidence intervals and *p*‐values were obtained by nonparametric bootstrap (5000 replicates). Hill pigeon crosses indicate (HP–HP), Feral pigeon crosses (FP–FP), Within‐hybrid crosses (LG–LG, F1–F1), and Heterotypic crosses (LG–F1, FP–F1, F1–LG). Δ*p* = Positive value indicates higher predicted mortality in cross A than group B, and negative Δ*p* indicates lower predicted mortality in A than B. OR > 1 indicates higher odds of mortality in A than B, OR < 1 indicates lower odds in A.

* Significant at *p* ≤ 0.05.

## 4 Discussion

### 4.1 Mating Patterns

We observed assortative mating within species (i.e., conspecific matings of hill or feral pigeons) (Table 1), despite the limited sample size of hill and feral pigeons. This homotypic pair formation is consistent with phenotypic preferences (e.g., for plumage, vocalization, or other mating signals). In feral pigeons, for example, pairings are more likely to form between individuals of similar body size, reflecting a size‐based assortative mating pattern (Johnston and Johnson 1989). This pattern has been interpreted through social dominance: larger individuals, with greater competitive ability and reproductive success, are preferentially chosen. Prior work has shown that feral pigeons are larger than hill pigeons across multiple morphological traits, with hybrids falling in between, underscoring the morphological distinctness among the three genetic ancestry classes (Kang et al. 2022). In the hybrid class (F1 and later‐generation), within‐class matings were rare and showed no significant deviation from expectation (Table 1; LG♂–LG♀: *Z* = 0.831; F1♂–F1♀: *Z* = 1.093). Matings with other classes occurred less frequently and showed a diverse pattern. Hybrid individuals exhibit an intermediate size between hill pigeons and feral pigeons, but their sizes are highly variable, suggesting that size may have influenced the observed results. Genetic similarity is likely a key factor influencing these assortative mating patterns (Emlen and Oring 1977). The observed homotypic matings indicate that individuals preferentially mate with genetically similar partners, driven by genetic compatibility, which facilitates the formation of breeding pairs that enhance offspring growth and survival.

### 4.2 Reproductive Isolation

Relative to hill × hill, neither hatchling nor fledgling mortality differed significantly in hybrid or heterotypic crosses, despite the high baseline hatchling mortality in hill × hill (Table 2; Figure 4). Relative to feral × feral, hatchling mortality was higher in both hybrid and heterotypic crosses (Table 2; Figure 4). At the fledging stage, elevated mortality was specifically associated with heterotypic crosses (Table 2; Figure 4). Observed patterns of reproductive mortality are consistent with stage‐specific postzygotic costs in crosses involving feral pigeons, which may indicate emerging reproductive isolation. Elevated mortality in within‐hybrid crosses is a feature of postzygotic isolation—barriers that act after fertilization—and is typically expressed as hybrid inviability or sterility, yielding reduced fitness relative to parental purebreds (Dobzhansky 1940; Muller 1942; Servedio and Noor 2003). Mechanistically, such mortality is plausibly driven by Dobzhansky–Muller–type genetic incompatibilities: alleles that are benign within the parental genomic backgrounds interact deleteriously in hybrids, disrupting development or physiology. Collectively, these findings provide evidence consistent with postzygotic barriers in crosses involving feral pigeons studied here.

### 4.3 Reinforcement

Relating our findings to reinforcement requires care. Classical reinforcement posits that reduced hybrid fitness selects for traits that decrease heterospecific crosses (Servedio and Noor 2003). In our system, we first quantified mating structure and then documented stage‐specific postzygotic costs—elevated hatchling mortality for hybrid and heterotypic crosses in feral anchored comparisons, and elevated fledgling mortality for heterotypic crosses (Table 2; Figure 4)—which together establish the preconditions for reinforcement rather than its completion. Accordingly, the mating patterns we observed—including assortative mating among cross types and the tendency for certain heterotypic pair types to occur more frequently than expected under random mating (Table 1; F1♂–LG♀: *Z* = 1.507; FP♂–F1♀: *Z* = 1.400)—are consistent with expectations under the hybrid‐swarm context documented for this system, in which the experimental cohort shows a hill‐skewed, continuous distribution of hybrid index (Figure 2). If reinforcement were already complete, heterotypic crosses would be strongly suppressed; instead, our results reveal a transitional structure typical of an ongoing hybrid swarm—strong assortative mating co‐occurring with residual, directional heterotypic crosses.

**FIGURE 5:**
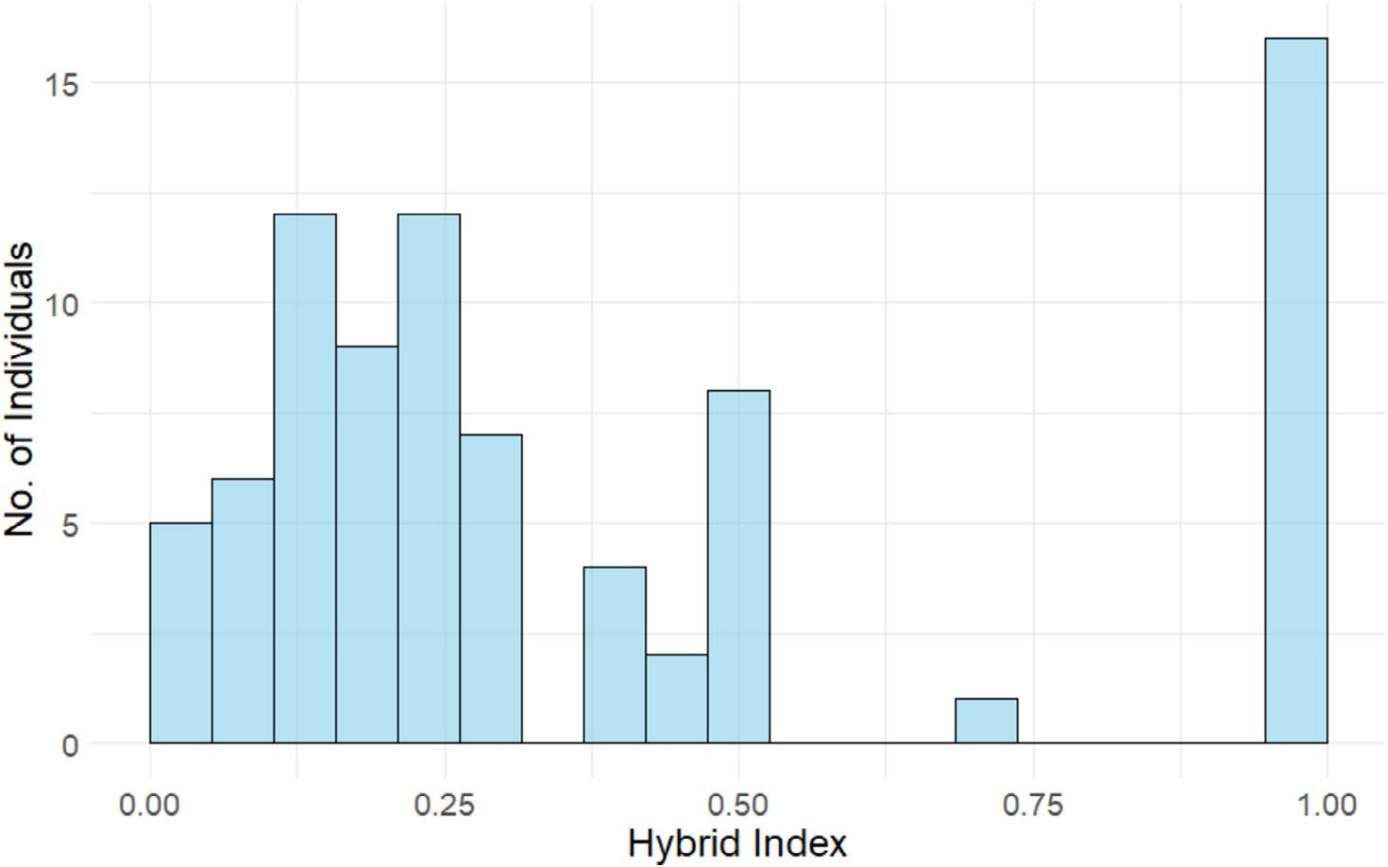
Distribution of hybrid index scores for experimental individuals (parents) and their offspring produced during the study.

Within this ongoing hybrid‐swarm system (Figure 2) and given the observed mating structure and reproductive outcomes (Table 1; Table 2; Figure 4), we outline expectations that jointly consider postzygotic isolation and the post‐reproduction shift in hybrid index scores (Figure 5; Table S1). Drawing on a density‐dependent perspective (Wood et al. 2021)—where hybridization tends to be negatively density‐dependent for the expanding taxon while competitive interactions intensify with density—we hypothesize that increasing feral representation in this mixed system may interact with the observed postzygotic costs to favor stronger conspecific mate choice in feral pigeons (Servedio and Noor 2003). In contrast, hill pigeons—showing weaker postzygotic costs in our data—may continue to form heterotypic crosses when conspecifics are scarce, sustaining introgression into the hill pigeons. Thus, although assortative mating predominates in hill pigeons, the persistence of some random mating and an apparent relaxation of postzygotic isolation, together with the potential strengthening of conspecific mate choice in feral pigeons, suggest ongoing introgression of feral ancestry into the hybrid population.

## Author Contributions

**Jin‐yong Kim:** conceptualization (equal), data curation (lead), formal analysis (lead), funding acquisition (equal), investigation (lead), methodology (lead), project administration (equal), resources (lead), software (lead), validation (lead), visualization (lead), writing – original draft (lead), writing – review and editing (lead). **Seung‐Gu Kang:** investigation (equal), resources (equal), writing – review and editing (equal). **Jongmin Yoon:** conceptualization (equal), project administration (equal), supervision (lead), validation (equal), writing – review and editing (equal).

## Funding

This work was supported by a grant from the National Institute of Ecology (NIE‐B2026‐46) and funded by the Ministry of Climate, Energy and Enviroment (MCEE) of the Republic of Korea.

## Ethics Statement

Our animal care and use protocol was also reviewed and approved by the IACUC at the National Institute of Ecology (permit number NIEIACUC‐2022‐014).

## Conflicts of Interest

The authors declare no conflicts of interest.

## Supporting information


**Table S1:** Multilocus hybrid marker dataset (HM30–HM59) and derived purity/hybrid indices for all individuals.

**Data S1:** ece373455‐sup‐0002‐DataS1.xlsx.

## Data Availability

All the required data are uploaded as Supporting Information.
