# Supplementary material for: Mating Patterns and Postzygotic Barriers in a Hybrid Swarm of Two Closely Related Pigeon Species
Source: Ecol Evol. 2026 Apr 15;16(4):e73455. doi: 10.1002/ece3.73455 (PMC13083217; doi:10.1002/ece3.73455)
Supplement: Supplementary file 1 — Table S1: Multilocus hybrid marker dataset (HM30–HM59) and derived purity/hybrid indices for all individuals. [file ECE3-16-e73455-s001.docx]

Table S1. Multilocus hybrid marker dataset (HM30–HM59) and derived purity/hybrid indices for all individuals

| **No.** | **Species** | **Color band** | **Metal band** | **Sex** | **HM30** | **HM37** | **HM41** | **HM43** | **HM45** | **HM55** | **HM56** | **HM59** | **Purity index** | **Hybrid index** | **Group** |
| --- | --- | --- | --- | --- | --- | --- | --- | --- | --- | --- | --- | --- | --- | --- | --- |
| 1 | Hybrid | GO/YA | 080-07122 | M | 0.5 | 1 | 1 | 1 | 1 | 1 | 1 | 1 | 0.9375 | 0.0625 | Parents |
| 2 | Hybrid | OBU/BUA | 080-07107 | F | 1 | 0.5 | 0.5 | 0.5 | 1 | 1 | 0.5 | 1 | 0.75 | 0.25 | Parents |
| 3 | Hybrid | GO/BUA | 080-05556 | F | 1 | 0.5 | 0.5 | 0.5 | 0.5 | 1 | 0.5 | 0.5 | 0.625 | 0.375 | Parents |
| 4 | Hybrid | GG/WA | 080-05558 | F | 1 | 0.5 | 1 | 1 | 1 | 1 | 1 | 1 | 0.9375 | 0.0625 | Parents |
| 5 | Hybrid | GW/BUA | 080-07106 | M | 0.5 | 1 | 1 | 0.5 | 1 | 1 | 1 | 1 | 0.875 | 0.125 | Parents |
| 6 | Hybrid | OY/BUA | 080-05559 | M | 1 | 0.5 | 1 | 0.5 | 1 | 1 | 1 | 1 | 0.875 | 0.125 | Parents |
| 7 | Hybrid | YO/OA | 080-05560 | M | 1 | 1 | 0.5 | 0.5 | 1 | 1 | 0.5 | 0.5 | 0.75 | 0.25 | Parents |
| 8 | Hybrid | BKBU/OA | 070-02062 | M | 1 | 1 | 0.5 | 1 | 1 | 1 | 0.5 | 1 | 0.875 | 0.125 | Parents |
| 9 | Hybrid | GBK/BKA | 070-02068 | M | 1 | 0.5 | 0.5 | 1 | 1 | 1 | 0.5 | 1 | 0.8125 | 0.1875 | Parents |
| 10 | Hybrid | GBK/GA | 070-02411 | M | 1 | 0.5 | 1 | 0.5 | 1 | 1 | 1 | 1 | 0.875 | 0.125 | Parents |
| 11 | Hybrid | GBK/WA | 070-02407 | M | 1 | 1 | 0 | 1 | 1 | 1 | 0 | 1 | 0.75 | 0.25 | Parents |
| 12 | Hybrid | GY/BUA | 070-02410 | M | 1 | 1 | 0.5 | 1 | 1 | 1 | 0.5 | 1 | 0.875 | 0.125 | Parents |
| 13 | Hybrid | BUW/OA | 070-02406 | M | 1 | 1 | 1 | 0.5 | 1 | 1 | 1 | 0.5 | 0.875 | 0.125 | Parents |
| 14 | Hybrid | GG/OA | 080-07120 | M | 1 | 0.5 | 0.5 | 1 | 1 | 1 | 0.5 | 1 | 0.8125 | 0.1875 | Parents |
| 15 | Hybrid | OG/OA | 080-07151 | F | 1 | 1 | 0.5 | 1 | 1 | 1 | 0.5 | 1 | 0.875 | 0.125 | Parents |
| 16 | Hybrid | WBU/WA | 070-02403 | F | 1 | 0.5 | 0.5 | 0.5 | 1 | 1 | 0.5 | 0.5 | 0.6875 | 0.3125 | Parents |
| 17 | Hybrid | GBU/BKA | 070-02404 | F | 1 | 0.5 | 1 | 1 | 1 | 1 | 1 | 1 | 0.9375 | 0.0625 | Parents |
| 18 | Hybrid | OG/BKA | 070-02405 | F | 1 | 0.5 | 1 | 1 | 1 | 1 | 1 | 1 | 0.9375 | 0.0625 | Parents |
| 19 | Hybrid | BKY/WA | 070-02408 | M | 0.5 | 1 | 0.5 | 0.5 | 1 | 1 | 0.5 | 1 | 0.75 | 0.25 | Parents |
| 20 | Hybrid | OBU/WA | 080-05566 | F | 1 | 0.5 | 0.5 | 1 | 1 | 1 | 0.5 | 1 | 0.8125 | 0.1875 | Parents |
| 21 | Hybrid | BUO/WA | 080-07152 | F | 1 | 1 | 0 | 1 | 1 | 1 | 0 | 1 | 0.75 | 0.25 | Parents |
| 22 | Hybrid | WO/GA | 070-00512 | M | 0.5 | 0.5 | 1 | 0.5 | 1 | 1 | 1 | 1 | 0.8125 | 0.1875 | Parents |
| 23 | Hybrid | YO/GA | 070-00510 | F | 0.5 | 1 | 0.5 | 1 | 1 | 1 | 0.5 | 1 | 0.8125 | 0.1875 | Parents |
| 24 | Hybrid | YG/OA | 070-02419 | M | 1 | 1 | 0.5 | 0.5 | 1 | 1 | 0 | 0.5 | 0.6875 | 0.3125 | Parents |
| 25 | Hybrid | YBK/BKA | 070-02420 | F | 1 | 1 | 1 | 0.5 | 1 | 1 | 1 | 0.5 | 0.875 | 0.125 | Parents |
| 26 | Hybrid | BKBU/WA | 070-02421 | F | 1 | 0.5 | 1 | 1 | 1 | 1 | 1 | 1 | 0.9375 | 0.0625 | Parents |
| 27 | Hybrid | BKY/OA | 070-02427 | M | 1 | 1 | 0.5 | 0.5 | 1 | 1 | 0.5 | 1 | 0.8125 | 0.1875 | Parents |
| 28 | Hybrid | GW/YA | 070-02428 | M | 1 | 0.5 | 0 | 1 | 1 | 1 | 0 | 1 | 0.6875 | 0.3125 | Parents |
| 29 | Hybrid | GG/GA | 070-02433 | M | 1 | 1 | 0.5 | 0.5 | 1 | 1 | 0.5 | 0.5 | 0.75 | 0.25 | Parents |
| 30 | Hybrid | BUO/BKA | 070-02434 | F | 1 | 0.5 | 1 | 1 | 1 | 1 | 1 | 1 | 0.9375 | 0.0625 | Parents |
| 31 | Hybrid | BUW/GA | 070-02061 | F | 1 | 1 | 1 | 0 | 0 | 1 | 1 | 0 | 0.625 | 0.375 | Parents |
| 32 | Hybrid | BUG/GA | 070-02063 | F | 1 | 1 | 1 | 1 | 1 | 0 | 1 | 1 | 0.875 | 0.125 | Parents |
| 33 | Hybrid | BUBK/YA | 070-02065 | F | 0.5 | 1 | 1 | 1 | 0.5 | 1 | 1 | 0.5 | 0.8125 | 0.1875 | Parents |
| 34 | Hybrid | BKW/GA | 070-02064 | M | 1 | 1 | 1 | 0.5 | 0.5 | 1 | 1 | 0.5 | 0.8125 | 0.1875 | Parents |
| 35 | F1 | YBK/BUA | 070-02066 | M | 0.5 | 0.5 | 0.5 | 0.5 | 0.5 | 0.5 | 0.5 | 0.5 | 0.5 | 0.5 | Parents |
| 36 | F1 | BUG/BKA | 070-02422 | M | 0.5 | 0.5 | 0.5 | 0.5 | 0.5 | 0.5 | 0.5 | 0.5 | 0.5 | 0.5 | Parents |
| 37 | F1 | BUW/BUA | 070-02439 | M | 0.5 | 0.5 | 0.5 | 0.5 | 0.5 | 0.5 | 0.5 | 0.5 | 0.5 | 0.5 | Parents |
| 38 | F1 | WW/GA | 070-02414 | M | 0.5 | 0.5 | 0.5 | 0.5 | 0.5 | 0.5 | 0.5 | 0.5 | 0.5 | 0.5 | Parents |
| 39 | F1 | BKBU/BUA | 080-05573 | M | 0.5 | 0.5 | 0.5 | 0.5 | 0.5 | 0.5 | 0.5 | 0.5 | 0.5 | 0.5 | Parents |
| 40 | F1 | BKG/WA | 070-02423 | F | 0.5 | 0.5 | 0.5 | 0.5 | 0.5 | 0.5 | 0.5 | 0.5 | 0.5 | 0.5 | Parents |
| 41 | F1 | GY/GA | 070-02437 | F | 0.5 | 0.5 | 0.5 | 0.5 | 0.5 | 0.5 | 0.5 | 0.5 | 0.5 | 0.5 | Parents |
| 42 | Hybrid | OBU/GA | 070-02402 | F | 1 | 0.5 | 1 | 0.5 | 1 | 0.5 | 1 | 0.5 | 0.75 | 0.25 | Parents |
| 43 | Feral | WW/OA | 070-00541 | M | 0 | 0 | 0 | 0 | 0 | 0 | 0 | 0 | 0 | 1 | Parents |
| 44 | Feral | OY/BKA | 070-00545 | F | 0 | 0 | 0 | 0 | 0 | 0 | 0 | 0 | 0 | 1 | Parents |
| 45 | Feral | WBK/GA | 070-00546 | F | 0 | 0 | 0 | 0 | 0 | 0 | 0 | 0 | 0 | 1 | Parents |
| 46 | Feral | BUO/OA | 070-00549 | M | 0 | 0 | 0 | 0 | 0 | 0 | 0 | 0 | 0 | 1 | Parents |
| 47 | Hill | GO/BKA | 070-02425 | F | 1 | 1 | 1 | 1 | 1 | 1 | 1 | 1 | 1 | 0 | Parents |
| 48 | Hill | BUBU/WA | 080-05853 | M | 1 | 1 | 1 | 1 | 1 | 1 | 1 | 1 | 1 | 0 | Parents |
| 49 | Hill | BUBU/BKA | 080-07101 | F | 1 | 1 | 1 | 1 | 1 | 1 | 1 | 1 | 1 | 0 | Parents |
| 50 | Hill | YO/BUA | 070-02442 | M | 1 | 1 | 1 | 1 | 1 | 1 | 1 | 1 | 1 | 0 | Parents |
| 51 |  |  | 070-02445 | M | 0 | 0 | 0 | 0 | 0 | 0 | 0 | 0 | 0 | 1 | Chick |
| 52 |  |  | 070-02446 | M | 0 | 0 | 0 | 0 | 0 | 0 | 0 | 0 | 0 | 1 | Chick |
| 53 |  |  | 070-02447 | M | 0 | 0 | 0 | 0 | 0 | 0 | 0 | 0 | 0 | 1 | Chick |
| 54 |  |  | 070-02458 | F | 0 | 0 | 0 | 0 | 0 | 0 | 0 | 0 | 0 | 1 | Chick |
| 55 |  |  | 070-02459 | F | 0 | 0 | 0 | 0 | 0 | 0 | 0 | 0 | 0 | 1 | Chick |
| 56 |  |  | 070-02467 | F | 0 | 0 | 0 | 0 | 0 | 0 | 0 | 0 | 0 | 1 | Chick |
| 57 |  |  | 070-02468 | M | 0 | 0 | 0 | 0 | 0 | 0 | 0 | 0 | 0 | 1 | Chick |
| 58 |  |  | 070-02470 | F | 0 | 0 | 0 | 0 | 0 | 0 | 0 | 0 | 0 | 1 | Chick |
| 59 |  |  | 070-02471 | M | 0 | 0 | 0 | 0 | 0 | 0 | 0 | 0 | 0 | 1 | Chick |
| 60 |  |  | 070-02480 | M | 0 | 0 | 0 | 0 | 0 | 0 | 0 | 0 | 0 | 1 | Chick |
| 61 |  |  | 070-02481 | F | 0 | 0 | 0 | 0 | 0 | 0 | 0 | 0 | 0 | 1 | Chick |
| 62 |  |  | 070-02482 | F | 0 | 0 | 0 | 0 | 0 | 0 | 0 | 0 | 0 | 1 | Chick |
| 63 |  |  | 070-02500 | M | 0.5 | 0.5 | 0.5 | 0 | 0 | 0.5 | 0.5 | 0 | 0.3125 | 0.6875 | Chick |
| 64 |  |  | 070-02496 | F | 0.5 | 1 | 0.5 | 0 | 0.5 | 0.5 | 0.5 | 0.5 | 0.5 | 0.5 | Chick |
| 65 |  |  | 070-02461 |  | 0.5 | 0.5 | 0.5 | 1 | 0 | 0.5 | 0.5 | 1 | 0.5625 | 0.4375 | Chick |
| 66 |  |  | 070-02497 | F | 1 | 0.5 | 0.5 | 0.5 | 0.5 | 0.5 | 0.5 | 0.5 | 0.5625 | 0.4375 | Chick |
| 67 |  |  | 070-02452 |  | 0.5 | 0.5 | 0.5 | 0.5 | 1 | 1 | 0.5 | 0.5 | 0.625 | 0.375 | Chick |
| 68 |  |  | 070-02487 | M | 1 | 1 | 0.5 | 0.5 | 0.5 | 0.5 | 0.5 | 0.5 | 0.625 | 0.375 | Chick |
| 69 |  |  | 070-02464 |  | 1 | 1 | 0.5 | 0.5 | 0.5 | 1 | 0.5 | 0.5 | 0.6875 | 0.3125 | Chick |
| 70 |  |  | 070-02484 | F | 1 | 0.5 | 0.5 | 0.5 | 1 | 1 | 0.5 | 0.5 | 0.6875 | 0.3125 | Chick |
| 71 |  |  | 070-02489 | F | 0 | 1 | 0.5 | 0.5 | 1 | 1 | 0.5 | 1 | 0.6875 | 0.3125 | Chick |
| 72 |  |  | 070-02493 | F | 0 | 0.5 | 1 | 0.5 | 0.5 | 1 | 1 | 1 | 0.6875 | 0.3125 | Chick |
| 73 |  |  | 070-02451 |  | 1 | 1 | 0.5 | 0.5 | 1 | 1 | 0.5 | 0.5 | 0.75 | 0.25 | Chick |
| 74 |  |  | 070-02460 |  | 0.5 | 0 | 1 | 1 | 0.5 | 1 | 1 | 1 | 0.75 | 0.25 | Chick |
| 75 |  |  | 070-02465 |  | 1 | 0.5 | 0.5 | 1 | 0.5 | 1 | 0.5 | 1 | 0.75 | 0.25 | Chick |
| 76 |  |  | 070-02495 | F | 1 | 1 | 0.5 | 0.5 | 1 | 1 | 0.5 | 0.5 | 0.75 | 0.25 | Chick |
| 77 |  |  | 070-02478 | F | 1 | 1 | 0.5 | 0.5 | 1 | 1 | 0.5 | 0.5 | 0.75 | 0.25 | Chick |
| 78 |  |  | 070-02483 | F | 1 | 0.5 | 0.5 | 1 | 1 | 1 | 0.5 | 1 | 0.8125 | 0.1875 | Chick |
| 79 |  |  | 070-02453 |  | 1 | 1 | 0.5 | 1 | 1 | 1 | 0.5 | 1 | 0.875 | 0.125 | Chick |
| 80 |  |  | 070-02474 | F | 1 | 1 | 0.5 | 1 | 1 | 1 | 0.5 | 1 | 0.875 | 0.125 | Chick |
| 81 |  |  | 070-02498 | F | 1 | 1 | 1 | 0.5 | 1 | 1 | 1 | 0.5 | 0.875 | 0.125 | Chick |
| 82 |  |  | 070-02477 | F | 1 | 1 | 1 | 1 | 1 | 1 | 1 | 1 | 1 | 0 | Chick |
